# Complete mitochondrial genome of the *Ptychobarbus chungtienensis* (Teleostei: Cyprinidae)

**DOI:** 10.1080/23802359.2016.1266916

**Published:** 2016-12-23

**Authors:** Yuping Qiu, Guozhu Chen

**Affiliations:** National Plateau Wetland Research Center, Southwest Forestry University, Kunming, Yunnan, China

**Keywords:** *Ptychobarbus chungtienensis*, Schizothoracinae, mitochondrial geneome, D-loop, tandem repeat

## Abstract

The complete mitochondrial genome of *Ptychobarbus chungtienensis* was first determined by using a PCR-based sequencing method. Its mitochondrial genome is 16,970bp in length, consisting of 13 protein-coding genes, 22 transfer RNA (tRNA) genes, 2 ribosomal RNA (rRNA) genes, and a non-coding control region, and its gene order was consistent with other fishes. In the control region, there was a 54 bp tandem repeat array identified at 3′ end. Phylogenetic analysis showed that *P. chungtiensensis* was clustered with other three species of *Ptychobarbus*.

*Ptychobarbus chungtienesis* is a freshwater fish species that belongs to subfamily Schizothoracinae (Teleostei: Cyprinidae), and is a Chinese endemic specie. As the threatening factors were increasing, the wild populations of *P. chungtienese* were declined, which resulted in this fish currently only distributed in Bita Lake in Yunnan Province of China and already considered as Endangered species. The schizothoracine fishes are the natural group of Cyprinidae family, and also are only taxon that have adapted to the natural environment of the Tibetan Plateau (He et al. 2004; He & Chen 2006; Yang et al. 2014).

The sample was captured from Bitahai wetland, Shangrila city, Diqing Prefecture, Yunnan Province, China (27°82′ N, 99°99′ E). Voucher specimens (No. 296) were deposited at the biological museum of Southwest Forestry University. We adopted PCR method to amplify the complete mitochondrial genome of the *P. chungtienesis*, and the PCR primers designed by ourselves according as the published sequence of Schizothoracinae fishes. The consensus sequence was employed by the DNASTAR7.1 tool and carefully checked. The complete mitochondrial genome of *P. chungtienesis* is 16,970bp (GeneBank Accession No. KY012741), consisting of 22 transfer RNA (tRNA) genes, 2 ribosomal RNA (rRNA) genes, 13 protein-coding genes, and a control region, showing 99% identities to the *P. kaznakovi* (GenBank: KM268050) (Ma et al. 2016). The order and direction of these genes were identical to other Schizothoracine fishes (Li et al. 2013; Qiao et al. 2014; Zhang et al. 2015). It’s worth mentioning that the D-loop of *P. chungtienesis* was 1161bp in length and 54bp tandem repeat array with the core repetitive sequence CACTTAATACTCTAACA-TTTTT-GACCAGCTAGCGTAGCTTAATATAAAATATAG was identified at 3′ end of D-loop. This tandem repeat in D-loop was also discovered in *P. Kaznakovi* (KM268050) from the same genus (Ma et al. 2016).

We used MEGA6 software (Tamura et al. 2013) to construct a maximum-likelihood tree (with 1000 bootstrap replicates) based on complete mitogenomes of 16 species from Schizothoracine subfamily and *Minytrema melanops* as an outgroup. The phylogenetic tree showed that four species from *Ptychobarbus* genus were clustered into a clade supported with high bootstrap value (Figure 1). The complete mitogenome of the *P. chungtienensis* in this study provides important data in evolutionary analysis of Schizothoracine phylogeny.

**Figure 1.**
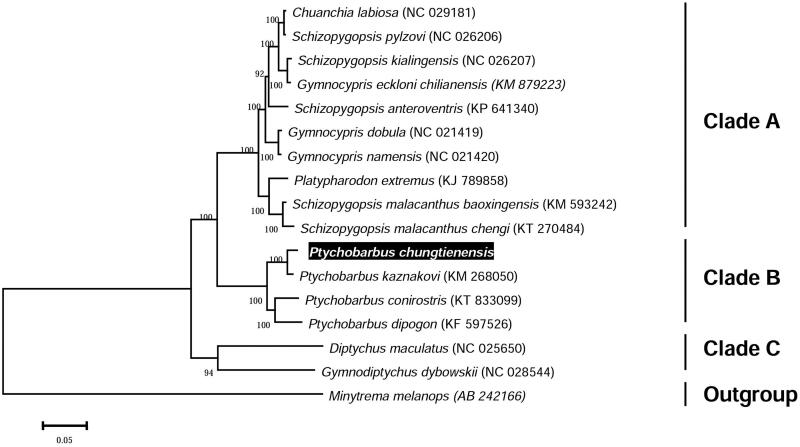
The maximum likelihood tree of *P. chungtienensis* and other species based on complete mitogenome. Bootstrap support is indicated at nodes. GenBank accession numbers are indicated in brackets.
